# Aguiar CA, Leister N, Bussadori JCC, Riesco MLG, Teixeira TT. Indicadores de monitoramento e avaliação dos Centros de Parto Normal Peri-hospitalares: resultados do estudo *Nascer nas Casas de Parto do Brasil.* Cad Saúde Pública 2025; 41(7):e00175024.

**DOI:** 10.1590/0102-311XER175024

**Published:** 2025-09-01

Onde se lê:

**Tabela 1:** Indicadores gerais.

Indicador	N Denominador	n Indicador	%	IC95%
Partos (Indicador 1a)	3.097	-	-	-
Admissões de adolescentes (Indicador 1c) *	3.111	292	9,4	2,1-14,6
Partos por enfermeiras/obstetrizes (Indicador 1b)	3.097	3.001	96,9	83,9-100,0
Partos por médicos (Indicador 1b)	3.097	96	3,1	0,0-16,1
Acompanhante (Indicador 7)	3.097	3.058	98,7	90,9-100,0
≥ 6 consultas pré-natais (Indicador 8) *	3.111	2.320	74,6	46,5-96,6
Avaliação ou procedimento médico (Indicador 12) **	2.512	6	0,2	0,0-0,7

IC95%: intervalo de 95% de confiança; CPNp: Centros de Parto Normal Peri-hospitalares.

Nota: itens 1a, 1b, 1c, 7, 8 e 12 da *Portaria nº 11/2015* (Anexo II) ^2^.

* Dados analisados de acordo com as admissões em 6 CPNp;

** Dados analisados de acordo com o total de partos em 7 CPNp.

Leia-se:

**Tabela 1:** Indicadores gerais.

Indicador	N Denominador	n Indicador	%	% mínimo-máximo
Partos (Indicador 1a)	3.097	-	-	-
Admissões de adolescentes (Indicador 1c) *	3.111	292	9,4	2,1-14,6
Partos por enfermeiras/obstetrizes (Indicador 1b)	3.097	3.001	96,9	83,9-100,0
Partos por médicos (Indicador 1b)	3.097	96	3,1	0,0-16,1
Acompanhante (Indicador 7)	3.097	3.058	98,7	90,9-100,0
≥ 6 consultas pré-natais (Indicador 8) *	3.111	2.320	74,6	46,5-96,6
Avaliação ou procedimento médico (Indicador 12) **	2.512	6	0,2	0,0-0,7

CPNp: Centros de Parto Normal Peri-hospitalares.

Nota: itens 1a, 1b, 1c, 7, 8 e 12 da *Portaria nº 11/2015* (Anexo II) ^2^.

* Dados analisados de acordo com as admissões em 6 CPNp;

** Dados analisados de acordo com o total de partos em 7 CPNp.

Onde se lê:

**Tabela 2:** Indicadores de assistência materna.

Indicador	N Denominador	n Indicador	%	IC95%
Amniotomia (Indicador 5) *	3.111	487	15,7	4,6-32,2
Ocitocina (Indicador 6) *	3.111	217	7,0	2,5-25,3
Partos na água (Indicador 1d) **	3.097	378	12,2	0,0-32,7
Partos verticais (Indicador 1e) ***	2.783	1.676	60,2	24,8-99,6
Períneo íntegro ***	2.783	896	32,2	14,9-46,6
Episiotomia (Indicador 2) **	3.097	11	0,4	0,0-3,1
Laceração 1º grau (Indicador 3) ***	2.783	1.234	44,3	25,6-59,9
Laceração 2º grau (Indicador 3) ***	2.783	608	21,8	11,3-50,6
Laceração 3º grau (Indicador 4) **	3.097	34	1,1	0,0-2,7
Laceração 4º grau (Indicador 4) **	3.097	2	0,1	0,0-0,2

IC95%: intervalo de 95% de confiança; CPNp: Centros de Parto Normal peri-hospitalares.

Nota: itens 1d, 1e, 2, 3, 4, 5 e 6 da *Portaria nº 11/2015* (Anexo II) ^2^.

* Dados analisados de acordo com as admissões em 6 CPNp;

** Dados analisados de acordo com o total de partos em 8 CPNp;

*** Dados relativos ao número de partos em 7 CPNp.

Leia-se:

**Tabela 2:** Indicadores de assistência materna.

Indicador	N Denominador	n Indicador	%	% mínimo-máximo
Amniotomia (Indicador 5) *	3.111	487	15,7	4,6-32,2
Ocitocina (Indicador 6) *	3.111	217	7,0	2,5-25,3
Partos na água (Indicador 1d) **	3.097	378	12,2	0,0-32,7
Partos verticais (Indicador 1e) ***	2.783	1.676	60,2	24,8-99,6
Períneo íntegro ***	2.783	896	32,2	14,9-46,6
Episiotomia (Indicador 2) **	3.097	11	0,4	0,0-3,1
Laceração 1º grau (Indicador 3) ***	2.783	1.234	44,3	25,6-59,9
Laceração 2º grau (Indicador 3) ***	2.783	608	21,8	11,3-50,6
Laceração 3º grau (Indicador 4) **	3.097	34	1,1	0,0-2,7
Laceração 4º grau (Indicador 4) **	3.097	2	0,1	0,0-0,2

CPNp: Centros de Parto Normal Peri-hospitalares.

Nota: itens 1d, 1e, 2, 3, 4, 5 e 6 da *Portaria nº 11/2015* (Anexo II) ^2^.

* Dados analisados de acordo com as admissões em 6 CPNp;

** Dados analisados de acordo com o total de partos em 8 CPNp;

*** Dados relativos ao número de partos em 7 CPNp.

Onde se lê:

**Tabela 3:** Indicadores neonatais.

Indicador	N Denominador	n Indicador	%	IC95%
Peso ao nascer < 2.500g (Indicador 9a) *	3.097	68	2,2	0,0-3,3
Peso ao nascer > 4.000g (Indicador 9a) *	3.097	67	2,2	0,8-4,8
RN com idade gestacional < 37 semanas (Indicador 9b) **	2.783	11	0,4	0,0-1,9
RN com idade gestacional > 41 semanas (Indicador 9b) **	2.783	53	1,9	0,0-8,5
Apgar < 7 no 5º minuto (Indicador 9c) *	3.097	18	0,6	0,0-1,3
Contato pele a pele imediato ao nascer (Indicador 9d) *	3.097	3.023	97,6	93,3-100

IC95%: intervalo de 95% de confiança; CPNp: Centros de Parto Normal Peri-hospitalares.

Nota: itens 9a, 9b, 9c, 9d da *Portaria nº 11/2015* (Anexo II) ^2^.

* Dados analisados de acordo com o total de partos dos 8 CPNp;

** Dados relativos ao número de partos em 7 CNPp.

Leia-se:

**Tabela 3:** Indicadores neonatais.

Indicador	N Denominador	n Indicador	%	% mínimo-máximo
Peso ao nascer < 2.500g (Indicador 9a) *	3.097	68	2,2	0,0-3,3
Peso ao nascer > 4.000g (Indicador 9a) *	3.097	67	2,2	0,8-4,8
RN com idade gestacional < 37 semanas (Indicador 9b) **	2.783	11	0,4	0,0-1,9
RN com idade gestacional > 41 semanas (Indicador 9b) **	2.783	53	1,9	0,0-8,5
Apgar < 7 no 5º minuto (Indicador 9c) *	3.097	18	0,6	0,0-1,3
Contato pele a pele imediato ao nascer (Indicador 9d) *	3.097	3.023	97,6	93,3-100,0

CPNp: Centros de Parto Normal Peri-hospitalares.

Nota: itens 9a, 9b, 9c, 9d da *Portaria nº 11/2015* (Anexo II) ^2^.

* Dados analisados de acordo com o total de partos dos 8 CPNp;

** Dados relativos ao número de partos em 7 CNPp.

Onde se lê:

**Tabela 4:** Indicadores relativos às transferências intraparto, pós-parto e neonatais e seus respectivos motivos.

Transferência e motivo	N Denominador	n Indicador	%	IC95%
Transferência intraparto * (Indicador 10)	3.367	584	17,3	6,0-53,5
Motivos das transferências intraparto **	2.283			
Solicitação de analgesia		105	4,6	0,0-9,7
Parada de progressão		82	3,6	2,3-5,5
Alteração dos batimentos cardíacos fetais		76	3,3	0,4-7,5
Líquido meconial		42	1,8	0,0-2,7
Bolsa rota prolongada		25	1,1	0,0-3,9
Pico hipertensivo ou alterações de sinais vitais		18	0,8	0,0-2,1
Solicitação materna		5	0,2	0,0-0,9
Apresentação anômala		2	0,1	0,0-0,2
Sangramento durante o trabalho de parto		2	0,1	0,0-0,2
Outros		18	0,8	0,0-1,4
Transferência pós-parto *** (Indicador 11a)	2.783	71	2,6	1,2-4,2
Motivos das transferências pós-parto ^#^	2.264			
Hemorragia pós-parto		24	0,9	0,0-1,2
Pico hipertensivo ou alterações de sinais vitais		11	0,4	0,0-1,2
Retenção placentária		9	0,3	0,0-0,6
Suspeita de infecção		6	0,2	0,0-1,2
Laceração 3º ou 4º grau		5	0,2	0,0-0,5
Anemia por hemorragia pós-parto		4	0,1	0,0-0,7
Hematoma perineal		2	0,1	0,0-0,2
Laceração cervical		2	0,1	0,0-0,2
Outros ^##^		3	0,1	0,0-0,5
Transferência neonatal ^#^ (Indicador 11b)	2.664	115	4,3	1,9-8,3
Motivos das transferências neonatais ^#^	2.664			
Desconforto respiratório		50	1,9	1,1-3,1
Icterícia precoce		20	0,8	0,0-1,4
Suspeita ou confirmação de infecção		16	0,6	0,0-1,2
Alterações cardíacas		8	0,3	0,0-0,9
Baixo peso		7	0,3	0,0-0,9
Hipoglicemia		5	0,2	0,0-0,5
Suspeita de alterações congênita		4	0,2	0,0-0,5
Outros ^###^		5	0,2	0,0-0,6

IC95%: intervalo de 95% de confiança; CPNp: Centros de Parto Normal Peri-hospitalares.

Nota: itens 10, 11a e 11b da *Portaria nº 11/2015* (Anexo II) ^2^.

* Dados relativos às admissões em 7 CPNp;

** Dados relativos às admissões em 4 CPNp;

*** Dados relativos aos partos ocorridos em 7 CPNp;

^#^ Dados relativos aos partos ocorridos em 6 CPNp;

^##^ Necessidade de avaliação de laceração perineal complexa de 2º grau, falta de energia elétrica no CPNp, paciente de alto risco transferida após admissão em expulsivo;

^###^ Febre, hipotermia, necessidade de reanimação, fratura de clavícula e reação vacinal.

Leia-se:

**Tabela 4:** Indicadores relativos às transferências intraparto, pós-parto e neonatais e seus respectivos motivos.

Transferência e motivo	N Denominador	n Indicador	%	% mínimo-máximo
Transferência intraparto * (Indicador 10)	3.367	584	17,3	6,0-53,5
Motivos das transferências intraparto **	2.283			
Solicitação de analgesia		105	4,6	0,0-9,7
Parada de progressão		82	3,6	2,3-5,5
Alteração dos batimentos cardíacos fetais		76	3,3	0,4-7,5
Líquido meconial		42	1,8	0,0-2,7
Bolsa rota prolongada		25	1,1	0,0-3,9
Pico hipertensivo ou alterações de sinais vitais		18	0,8	0,0-2,1
Solicitação materna		5	0,2	0,0-0,9
Apresentação anômala		2	0,1	0,0-0,2
Sangramento durante o trabalho de parto		2	0,1	0,0-0,2
Outros		18	0,8	0,0-1,4
Transferência pós-parto *** (Indicador 11a)	2.783	71	2,6	1,2-4,2
Motivos das transferências pós-parto ^#^	2.264			
Hemorragia pós-parto		24	0,9	0,0-1,2
Pico hipertensivo ou alterações de sinais vitais		11	0,4	0,0-1,2
Retenção placentária		9	0,3	0,0-0,6
Suspeita de infecção		6	0,2	0,0-1,2
Laceração 3º ou 4º grau		5	0,2	0,0-0,5
Anemia por hemorragia pós-parto		4	0,1	0,0-0,7
Hematoma perineal		2	0,1	0,0-0,2
Laceração cervical		2	0,1	0,0-0,2
Outros ^##^		3	0,1	0,0-0,5
Transferência neonatal ^#^ (Indicador 11b)	2.664	115	4,3	1,9-8,3
Motivos das transferências neonatais ^#^	2.664			
Desconforto respiratório		50	1,9	1,1-3,1
Icterícia precoce		20	0,8	0,0-1,4
Suspeita ou confirmação de infecção		16	0,6	0,0-1,2
Alterações cardíacas		8	0,3	0,0-0,9
Baixo peso		7	0,3	0,0-0,9
Hipoglicemia		5	0,2	0,0-0,5
Suspeita de alterações congênita		4	0,2	0,0-0,5
Outros ^###^		5	0,2	0,0-0,6

CPNp: Centros de Parto Normal Peri-hospitalares.

Nota: itens 10, 11a e 11b da *Portaria nº 11/2015* (Anexo II) ^2^.

* Dados relativos às admissões em 7 CPNp;

** Dados relativos às admissões em 4 CPNp;

*** Dados relativos aos partos ocorridos em 7 CPNp;

^#^ Dados relativos aos partos ocorridos em 6 CPNp;

^##^ Necessidade de avaliação de laceração perineal complexa de 2º grau, falta de energia elétrica no CPNp, paciente de alto risco transferida após admissão em expulsivo;

^###^ Febre, hipotermia, necessidade de reanimação, fratura de clavícula e reação vacinal.

